# Application of MXene in the diagnosis and treatment of breast cancer: A critical overview

**DOI:** 10.3389/fbioe.2022.984336

**Published:** 2022-08-24

**Authors:** Sara Ranjbari, Mahdieh Darroudi, Behnaz Hatamluyi, Reza Arefinia, Seyed Hamid Aghaee-Bakhtiari, Majid Rezayi, Majid Khazaei

**Affiliations:** ^1^ Chemical Engineering Department, Faculty of Engineering, Ferdowsi University of Mashhad, Mashhad, Iran; ^2^ Department of Physiology, Faculty of Medicine, Mashhad University of Medical Science, Mashhad, Iran; ^3^ Department of Medical Biotechnology and Nanotechnology, School of Science, Mashhad University of Medical Science, Mashhad, Iran; ^4^ Department of Pharmacology, Faculty of Medicine, Mashhad University of Medical Sciences, Mashhad, Iran; ^5^ Medical Toxicology Research Center, Mashhad University of Medical Science, Mashhad, Iran; ^6^ Metabolic Syndrome Research Center, Mashhad University of Medical Science, Mashhad, Iran

**Keywords:** biosensor, biomedicine, advanced nanomaterials, biomedical analysis, cancer treatment, breast cancer, MXene

## Abstract

Breast cancer is the second most common cancer worldwide. Prognosis and timely treatment can reduce the illness or improve it. The use of nanomaterials leads to timely diagnosis and effective treatment. MXenes are a 2D material with a unique composition of attributes, containing significant electrical conductance, high optical characteristics, mechanical consistency, and excellent optical properties. Current advances and insights show that MXene is far more promising in biotechnology applications than current nanobiotechnology systems. MXenes have various applications in biotechnology and biomedicine, such as drug delivery/loading, biosensor, cancer treatment, and bioimaging programs due to their high surface area, excellent biocompatibility, and physicochemical properties. Surface modifications MXenes are not only biocompatible but also have multifunctional properties, such as aiming ligands for preferential agglomeration at the tumor sites for photothermal treatment. Studies have shown that these nanostructures, detection, and breast cancer therapy are more acceptable than present nanosystems in *in vivo* and *in vitro*. This review article aims to investigate the structure of MXene, its various synthesis methods, its application to cancer diagnosis, cytotoxicity, biodegradability, and cancer treatment by the photothermal process (*in-vivo* and *in-vitro*).

## Graphical Abstract

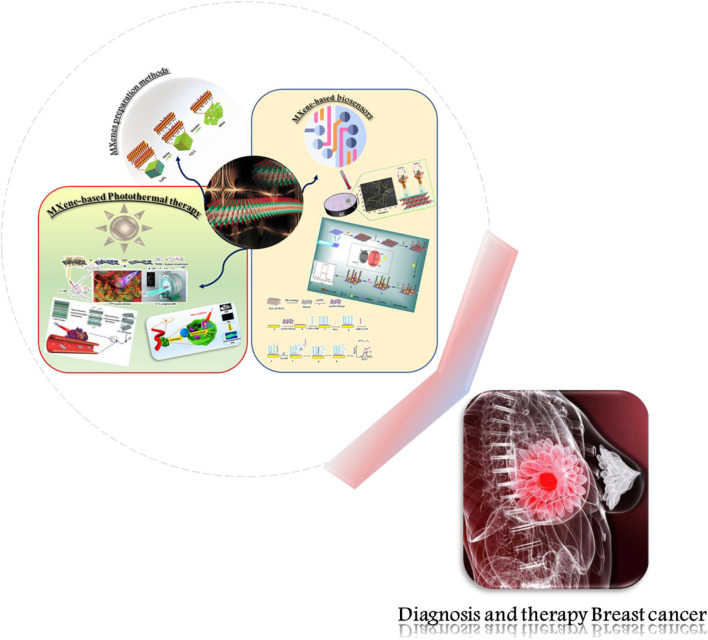

## 1 Introduction

According to the World Health Organization, breast cancer has the second-highest prevalence of cancer worldwide, with nearly two million breast cancers diagnosed in 2018 (Mittal et al., 2017; Senel et al., 2019; Nazari et al., 2021). Like other cancers, breast cancer occurs when breast cells begin to grow out of control (Waks and Winer, 2019). A few changes in the nipple and discoloration of the breast can be symptoms of breast cancer. Also, cancer cells are found mainly in the breast and the lymph nodes in the armpit and armpit (Becker, 2015; Waks and Winer, 2019). Stages 1–4 are dedicated to breast cancer, depending on where the tumor is found. In stage 4, metastatic breast cancer, cells have spread to other places in the body away from the mammary and axillary lymph nodes (Waks and Winer, 2019).

According to different types of proteins in a cell, this type of cancer is classified into three types. 70% of breast cancers involve Hormone-positive receptors that have a progesterone receptor (PR) or estrogen receptor (ER) on the cancer cell (Waks and Winer, 2019). About 15%–20% of breast cancers involve the HER2 + receptor, now known as ERBB2 +, and about 15% of breast cancers are triple-negative and do not have ER, PR, or ERBB2 protein in the cancer cells. The prognosis and therapy of this cancer depend on the type of cancer and its stage (Waks and Winer, 2019). Primarily, breast cancer therapy includes medications, chemotherapy, radiation, and surgery to remove cancer cells from the human body (DeSantis et al., 2019; Tabadkani et al., 2021). Because multiple agents are implicated at the beginning of cancer, these agents can show different signs depending on the kind and place of the tumor. Consequently, the remedy requires early diagnosis, efficient therapy methods, and post-remedy care to prevent relapse (Ali et al., 2015).

Today, nanoparticles such as Au, Ag, CNT, graphene oxide, QDs, MXene, etc., are used in biomedicine and cancer treatment (Wang et al., 2021a; Darroudi et al., 2021; Ma et al., 2022). Nanotechnology study, design, and fabrication of nanoscale materials or machines with petite lengths (10^–9^ m) are helpful for various applications. The nanoparticles’ properties differ from bulk materials due to their excellent surface area and small dimensions. The chemical, physical, optical, and electronic material properties change with the shape, area, and size of the particles that make them up. These excellent features allow them to show outstanding performance in diagnosing and/or efficient treating various diseases, such as cancer, based on fine-tuning their morphology, surface characteristics, and size (Salata, 2004; Majeed et al., 2019; Qi et al., 2019). MXenes (transfer metal carbides), as 2D (two dimensions) materials, have broad properties such as extensive surface area, high conductivity, and excellent photothermal conversion yield, along with powerful absorption in the NIR area (near-infrared) (Rasool et al., 2016; Pandey et al., 2018; George and Kandasubramanian, 2020). MXenes can be used in a wide range of medical fields like drug delivery (Han et al., 2018; Zhang et al., 2020; Zhu et al., 2021), biomedicine, cancer treatment (Lin et al., 2017; Yu et al., 2017), anti-bacterial (Rasool et al., 2016; Jastrzębska et al., 2019), and diagnosis (Lin et al., 2018; Shurbaji et al., 2021).

This review article aims to examine the structure of MXene, various methods of its synthesis, and its application to cancer diagnosis and cancer treatment by the photothermal process (*in-vivo* and *in-vitro*). In section, the photothermal process addressed the issues of cytotoxicity and biodegradability.

## 2 MXenes preparation methods

**FIGURE 1:**
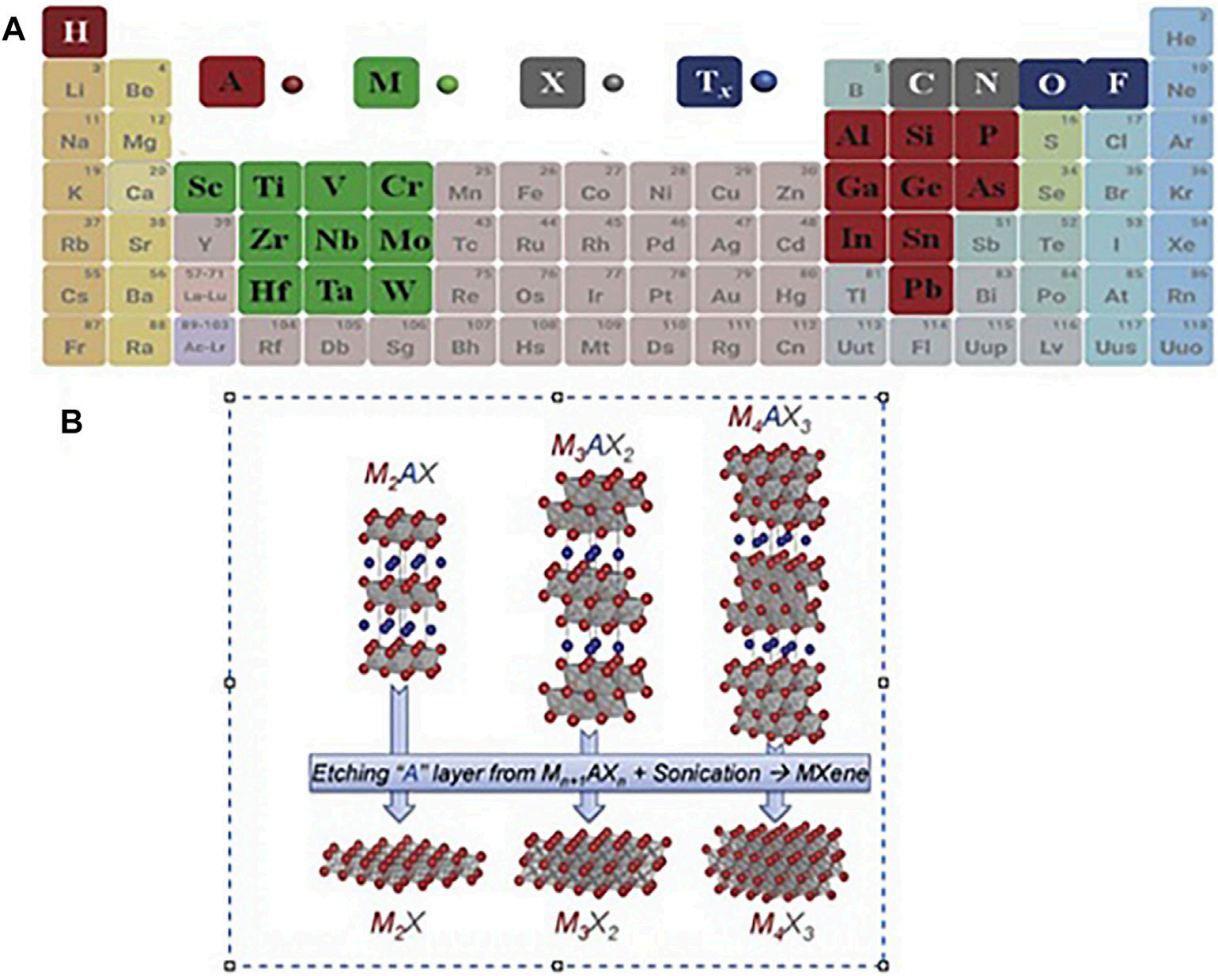
**(A)** Periodic table of the elements utilized from MAX phases (Wang et al., 2018), **(B)** Image of etching layers A from the corresponding MAX phases (Naguib et al., 2014).

Materials such as graphene with a 2D (Two-dimensional) layer structure have been noted in their particular structure (Tang et al., 2013). High surface area, functional surface, electrical conductivity (Karlsson et al., 2015), and optical properties (Nicolosi et al., 2013). In 2011 Naguib and Gogotsi et al. (2015) at Drexel University discovered 2D Ti_3_C_2_ powder (Titanium carbide), the MXene household’s first candidate (Naguib et al., 2011a; Naguib et al., 2014). MXenes have unique structural and electronic features, making them one of the eldest families of two-dimensional materials used for different applications (Sobolčiak et al., 2019). These materials are of the chemically etched metal carbonitrides and carbides, which have the generic formula M_n+1_X_n_T_x_, whereas M refers to Mn, V, Cr, Hf, Ti, Nb, Zr, Sc, Wd etc., N or C; n is one or two; Tx refers to oxygen, hydroxyl, or fluorine MXenes be prepared by etching (Cd, Ga, Si, As, Al, Ge, In, Ti, and Sn elements) layers of MAX phase. Figure 1A of the periodic table shows that using the ingredients of MAX phases, M_n+1_AX_n_ (MAX) phases are usually the starting compounds. As shown in Figure 1B, MXenens are formed by exfoliating in the A (Cd or Al) layers (Guo et al., 2016; Wang et al., 2018; Hart et al., 2019).

In general, there are two methods for synthesizing 2D substances. The bottom-up is the first method. For example, CVD (chemical vapor deposition) would generate great definition films in different layers. This way is not commonly used to synthesize MXenes, since the resulting films are not a single layer. Xu et al. (2015) utilized the CVD method to make Molybdenum carbide (Mo_2_C), Tungsten carbide, and Tantalum carbide thin films. However, even the thinnest Mo_2_C films had a minimum of six Mo_2_C layers and not single MXenes sheets. The second method is a top-down method that involves peeling off layered solids. The second method would be classified into two types mechanical and chemical peels.

For instance, an adhesive can use an adhesive strip to detach graphene layers (Novoselov et al., 2004). This method is not appropriate for MAX phases because, compared to another three-dimensional solid utilized as precursors to their two-dimensional similar, the bonds among M -Al are, in Most cases, metallic/covalent. Most MAX steps based on Al are synthesized to above T = 1,300°C (Barsoum, 2013). This approach is not applicable here. The remarkable point is that before the discovery of MXene, it was thought that only weakly bonded three-dimensional layered solids could be delaminated. Therefore, the top-down method converts three-dimensional to two-dimensional solids by chemical peeling by weakening the interlayer bonds. One way is to bond the layers together to be easily dispersed in a solvent (Nicolosi et al., 2013). Therefore, the vital issue is to find the right combination of intercalant and solvent. Chemical etching is the first method to synthesize Mxenes from MAX phases (bonded solids) (Naguib et al., 2011b; Li et al., 2015). At the moment, various types of ternary carbide and MAX nitride have been mentioned, which make a significant difference to this family. According to theoretical predictions, more than thirty types of MXenes have been tested; more are expected to be used (Pan et al., 2017; Frey et al., 2019). The various MXene acquired to date have been synthesized using different methods, precursors, etching methods, and bright lights (Garg et al., 2020).

Precursors, MAX phases form a big family of 130 or more combinations, most of which are crystallized in the space group P63/mmc, or derived. This structure is combined with MX6 octagonal combined with net layers of A. The principal variation between the three types (n is one, two, or three) of the MAX phase is the number of layers M (2–4) between layers A.

In the formation of MXene from MAX, etched layers are replaced by various groups of Tx like fluorine and oxygen. After the etching process, the material is composed of M_n+1_X_n_T_x_ in several layers, the bond between which is hydrogen and Vander Waals (Verger et al., 2019).

Exfoliation is after the etching stage. Exfoliation hinges on the etching methods and the position of the MXene. Removed by-products (such as Aluminum fluoride), the resulting layers were rinsed multiple times with water after the etching process. The acid may be utilized for pre-washing with H_2_SO_4_ or hydrochloric acid as a salt dissolving aid (aluminum fluoride or lithium fluoride). Only then can the layers be exfoliated to form colloidal suspensions containing several or more layers of MXene (Naguib et al., 2011b; Ghidiu et al., 2016; Urbankowski et al., 2016). Table 1 summarizes the different types of MXene synthesized methods.

**TABLE 1:** Various types of MXenes synthesis methods.

Types of MXenes	Methods	Application	Refs
Ti_3_C_2_Tx	Acid (HCl + LiF)	Direct absorption solar collectors	Li et al. (2020)
Ti_3_C_2_Tx	Acid	Heterogeneous catalysts	Zeng et al. (2021)
	HF		
Ti_3_C_2_Tx	Acid (HCl + LiF)	Flexible Supercapacitors	Sun et al. (2021)
Ti_3_C_2_Tx	Acid (HCl + LiF)	Adsorption	Ihsanullah and Ali, (2020)
Ti_3_C_2_Tx	Acid (HCl + LiF)	Adsorption	Khan et al. (2019)
Ti_3_C_2_	Acid (NH_4_HF_2_)	electrocatalyst	Abdullah et al. (2020)
Ti_3_C_2_Tx	Acid	Gas barrier nanocomposite films	Woo et al. (2020)
	HF		
Ti_3_C_2_	Acid (NH_4_HF_2_)	Energy storage properties and thermal conductivity	Aslfattahi et al. (2020)
Ti_2_NTx	Acid	Biological activity	Szuplewska et al. (2019b)
	HF		
V_2_CTx	Acid	Aluminum Batteries	VahidMohammadi et al. (2017)
	HF		
Ti_2_N	Acid	Surface-Enhanced Raman Scattering Substrate	Soundiraraju and George, (2017)
	HF		
Ti_3_C_2_Tx	NH_3_F	supercapacitors	Wang et al. (2016)
Zr_3_C_2_Tx	Acid	Electrical energy storage	Zhou et al. (2016)
	HF		
Ti_3_C_2_Tx	Acid (HCl + LiF)	Electrochemical sensor	Wang et al. (2021c)
Ti_3_C_2_Tx	NaOH	—	Li et al. (2018)
Ti_4_N_3_	Molten salts	—	Urbankowski et al. (2016)
(Mo_2_Ti_2_)C_3_Tx	TBAOH + HF	Thermoelectricity	Anasori et al. (2016)

## 3 Biosensors for breast cancer diagnosis

As previously mentioned in the first part of this article, various diagnosis methods of breast cancer based on Mxene were surveyed. Also, the advantage and limitations of these materials were investigated. In summary, ultrasound imaging, ELISA, IHC, and mammography are usually utilized for breast cancer detection and monitoring its advance (Ali et al., 2015). Nevertheless, each one of these diagnostic methods faces constraints such as specimen pretreatment, the need for expensive equipment, time-consuming, etc. Thus, it is expected to use novel, sensitive, rapid, and less aggressive methods such as biosensors to detect breast cancer (Dervisevic et al., 2020; Wang Z. et al., 2021).

**FIGURE 2:**
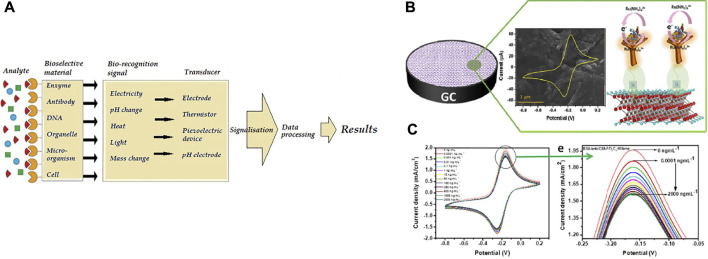
**(A)** The biosensor processes principle (Kivirand et al., 2013) **(B)**Schematic of the electrochemical biosensor (BSA/anti-CEA/f-Ti_3_C_2_-MXene/GCE) for CEA diagnosis, **(C)** diagram of electrochemical biosensor replies at various concentrations of CEA (Kumar et al., 2018).

Each biosensor is applicably comprised of three parts. The first section of the biosensor is the biological component that is reliable for analyte detection and causing the answer signal. The signal caused is then converted, which is reliable for analyte detection and causing the answer signal. The signal caused is then converted into a recognizable response by a second part called a transducer, the most vital part of any biosensor system. The third section is the biosensor detector, which processes and amplifies the signals before display utilizing an electronic display technique (Kivirand et al., 2013; Parkhey and Mohan, 2019). The different stages in signal processing of a biosensor, from measurement to transmission to display, are illustrated in Figure 2A.

Biosensors could be organized by conforming to the bio-identification element or physiochemical transfer type. Biosensors could be classed as piezoelectric, electrochemical, thermal and optical biosensors based on the converter (Pejcic et al., 2006; Darroudi et al., 2022; Hatamluyi et al., 2022).

Among biosensor types, Electrochemical biosensors are the widest biosensors studied because they have the boon of low detection limit, the plainness of construction, specificity, and comfort of the procedure. With recent boons in electronic tools, the biosensors cloud be used as on-chip laboratory instruments for *in-vivo* monitoring or as a handheld technology for on-site miniature (Ronkainen et al., 2010; Sawant, 2017).

### 3.1 MXene-based biosensors

Kumar et al. (2018) designed an unlabeled and high-sensitivity electrochemical biosensor for carcinoembryonic antigen (CEA) diagnosis based on Ti_3_C_2_ nanosheets. Then Ti_3_C_2_ nanosheets were functionalized with APTES for anti-CEA covalent stabilization. Figure2B shows A schematic of the electrode surface and the redox probe interplay. The designed biosensor (BSA/anti-CEA/f-Ti_3_C_2_-MXene/GCE) shows a wide detection range of 0.0001–2000 ng ml^−1^ (Figure 2C) with LOD 0.000018 ng ml^−1^.

**FIGURE 3:**
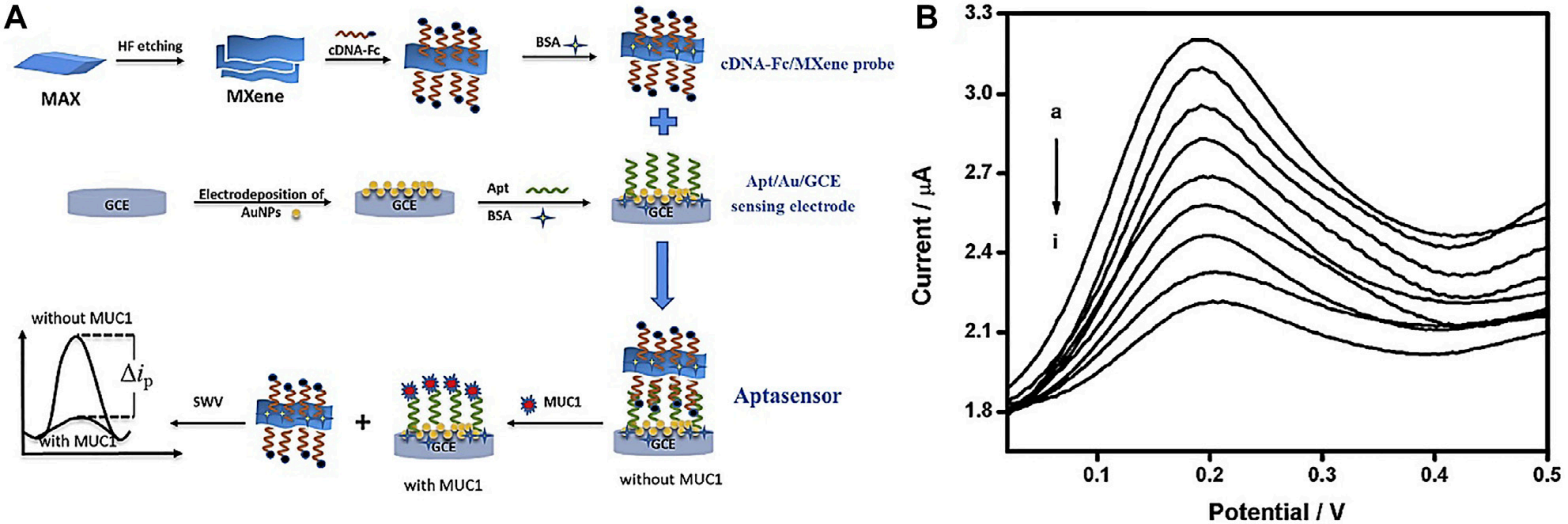
**(A)** Schematic of competitive aptasensor manufacturing and detection method, **(B)** diagram of electrochemical biosensor replies at various concentrations of MUC1 (Wang et al., 2020).

In another study, Wang et al. (2020) developed a competitive electrochemical biosensor-based cDNA-Fc/MXene probe to detect the MUC1 (Mucin1) as a breast cancer marker. MUC1 is a transmembrane glycoprotein, which is attention due to its unnormal expression in tumor tissues (people patients) for detection (Li et al., 2019). MXene was used as a nanobearer for cDNA-Fc to strengthen diagnosis signals and provided wide connection locations for cDNA-Fc binding. Figure 3A shows that detection involves three operations: linking the cDNA-Fc over MXene, bonding between Apt over the Au/GCE, and competitive detection of MUC1. To detect MUC1, the cDNA-Fc/MXene probe binds with Apt/Au/GCE and forms the aptasensor cDNA-Fc/MXene/Apt/Au/GCE. next stage; the aptasensor was registered in phosphate buffer solution as the primary signal. When the aptasensor is utilized to detect MUC1, the competitive approach begins. The MUC1 vies with cDNA-Fc/MXene probe for connecting the Apt/Au/GCE. Linking of MUC1 to aptamer causes a change in DNA composition, forcing the previously formed Apt/cDNA-Fc double strand to disintegrate and the cDNA-Fc/MXene probe detached from the biosensor, reducing the signal. The designed electrochemical aptasensor offers a large linear range from 1.0 p.m. to 10 μM (Figure 3B) and a LOD of 0.33 p.m., which is a bright idea in clinical detection.

**FIGURE 4:**
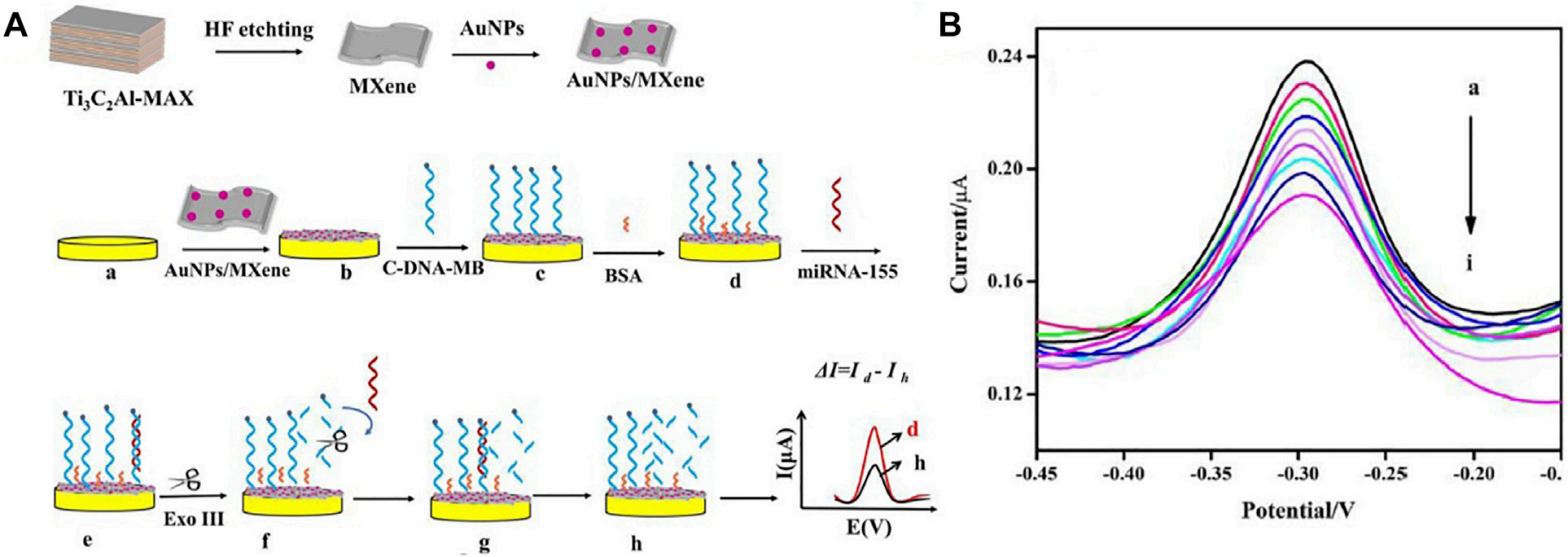
**(A)** Schematic of manufacturing electrochemical biosensor based Au/MXene to diagnosis miRNA-155, **(B)** diagram of electrochemical biosensor replies at various concentrations of miRNA-155 (linear range from 1.0 fM −10 nm) (Yang et al., 2020).

Interestingly, Yang et al. (2020) developed an electrochemical biosensor (cDNA/AuNPs/MXene) for mir-155 diagnosis by cascading target recovery using exonuclease III (Figure 4A). The biosensor 3D structure of the AuNPs/Ti3C2 enjoys significant electrical conductance, wide integrated surface area, and electrocatalytic attributes. Au nanoparticles are utilized to stabilize adsorbed cDNA by Au-S chemical bonding. The cDNA was bonded together with MB then the primitive DPV signal was recorded (Id). Next, cDNA and miRNA-155 were connected. Then exonuclease III recapitulated the end of the 3 ′cDNA in a double-stranded format, causing a reduced electrochemical signal (Ih). The designed electrochemical biosensor achieved a wide linear range from 1.0 fM -10 nm (Figure 4B) and a LOD of 0.35 fM. It also shows reproducibility, stability, and desirable characteristics.

**FIGURE 5:**
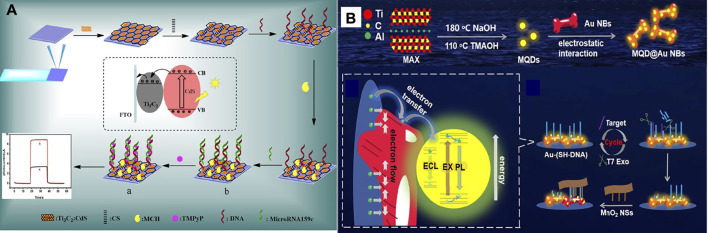
**(A)** Schematic of manufacturing photoelectrochemical biosensor based a Ti_3_C_2_:CdS to detect miRNA159c (Liu S.-T. et al., 2020), **(B)** Schematic of manufacturing ECL biosensor based on MQD/Au to detect miRNA-26a (Nie et al., 2021).

In another study, Liu et al. (2020) designed a photoelectrochemical biosensor based on a Ti_3_C_2_:CdS to detect miRNA159c (Figure 5A). Nanocomposites of CdS: Ti_3_C_2_ were utilized as materials of optoelectronic, which remarkably improved the photoelectric transformation yield. The linear range of miRNA159c was 1.0 × 10^–6^ to 1.0 × 10^–13^ mol L^−l,^ and the LOD of 33 fmol L^−l^. The designed biosensor provided an adequate diagnosis for breast cancer. Also, Nie et al. (2021) manufactured an Electrochemiluminescence biosensor based on MXene-quantum dot (MQD) and gold-nano bone detection of miRNA-26a, as shown in Figure 5B. The green procedure synthesized MQDs. MQDs and gold NBs with unparalleled electrochemical effects significantly increased electrochemiluminescence with conductivity and SPR properties. As a result, the diagnosis concentration was wide-ranging from 5 fM-10 nm and the LOD was 1.7 fM. This biosensor has been used successfully to diagnose serum samples from patients of clinical.

As can be seen, all studies with a variety of biomarkers for detecting breast cancer using MXene showed a better detection limit and range. In the following, the detection range and limit of detection of different types of MXene-based biosensors for breast cancer diagnosis have been brought in Table 2.

**TABLE 2:** The types of MXene-based biosensors for detecting breast cancer.

Target	Nanoparticle	Type of biosensor	Linear range/LOD	Refs
miRNA-26a	MQD@Gold	Electrochemiluminescence	Linear range = 5 fM to 10 nM	Nie et al. (2021)
			LOD = 1.7 fM	
MUC1	MXene/Au	Electrochemical aptasensor	Linear range = 1.0 pM–10 μM LOD = 0.33 p.m.	Wang et al. (2020)
miRNA-155	AuNPs/Ti_3_C_2_ MXene	Electrochemical biosensor	linear range = 1.0 fM to 10 nM	Yang et al. (2020)
			LOD = 0.35 fM	
CEA	Ti_3_C_2_-MXene	Electrochemical biosensor	Linear range = 0.0001–2000 ng ml^−1^	Kumar et al. (2018)
			LOD = 0.000018 ng ml^−1^	
microRNA159c	Ti_3_C_2_:CdS	Photoelectrochemical biosensor	Linear range = 1.0* 10^–6^–1.0 * 10^–13^ mol L^−l^	Liu et al. (2020a)
			LOD = 33 fmol L^−1^	

## 4 Systemic therapy considerations

Breast cancer is mainly removed via surgery with radiation therapy or chemotherapy (Nounou et al., 2015). Though these are the most practical therapies, they are by an imperfect delete of tumor via surgery that can conduce to tumor relapse (Tohme et al., 2017). In addition, radiation therapy and chemotherapy have multiple side effects such as problems of intestinal, harm to healthy tissues, nausea, and loss of hair (Hussein et al., 2019). Photothermal therapy (PTT) is a non-invasive treatment of cancer that murders tumor cells with heat and may altogether remove the tumor (Gong et al., 2020; Jiang et al., 2020; Wang et al., 2021b). Accordingly, it is more premier compared to removing surgically.

### 4.1 Biocompatibility and toxicity

Williams defined biocompatibility as the biomaterial’s ability to accomplish its intended function concerning medical treatment without causing any local or systemic adverse on the receptor or beneficiary of that treatment, but the most appropriate helpful tissue or cellular reply in that particular situation and optimizing the clinically related performance of that treatment (Williams, 2008). Biomaterial biocompatibility is fundamental system property emanated from medical, physical, biological, chemical, and design elements (Ahmed et al., 2012).

Therefore, materials of biocompatible requirements to have the feature such as suitable mechanical loading necessary; capacity for long-time storage, against chemical assault resistance by physiological fluids, resistance to corrosion, suitable density, not cause allergic or immunologic responses, and no poisonous or carcinogenic; etc. Toxicological effects of NPs relate to their ability to adversely affect human or animal physiology or directly interfere with organ and tissue function. The overall shape, particle size, surface charge, stability, and composition of NPs, all play a role in toxicity. As these nanoparticles are used in biomedical applications, they will be directly in contact with tissues and cells, making their biocompatibility a vital issue (Li et al., 2012).

The next part examines the photothermal property, biocompatibility, and toxicity of MXene nanoparticles in terms of compatibility, tissue compatibility, and cytotoxicity.

### 4.2 Photothermal therapy

MXene has a photothermal efficacy, meaning that it could transform the energy of Laser- light into the power of heat by intensifying the surface plasmon efficacy. Thus, scientists have researched MXenes for the PTT of cancer, who used in the murdering of cancer tumors via heat, which leads to denaturation of protein and, finally, cell death (Liu et al., 2017; Liu et al., 2018a; Wang and Cheng, 2019). MXenes with a size of about 180 nm could attain the cancerous microenvironment via increasing permeance and maintaining EPR (Gazzi et al., 2019).

**FIGURE 6:**
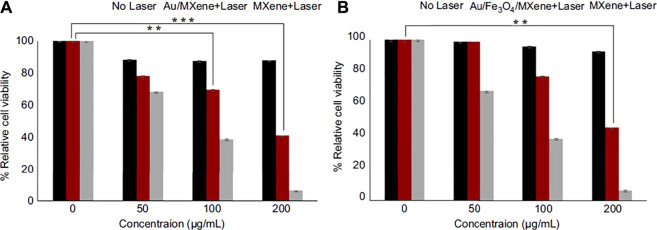
The relative viability of MCF7 cells since incubation (various concentrations) **(A)** Au/MXene and **(B)** Au/Fe_3_O_4_/MXene and Ti_3_C_2_ (MXene) following by NIR 808 nm laser radiance with a might density of 1.0 W/cm^2^ for 5 min Standardized to control (without laser treated cells) (Hussein et al., 2019).

For example, Hussein and colleagues (Hussein et al., 2019) designed plasmonic-based nanocomposites Au/Fe_3_O_4_/Ti_3_C_2_ and Au/Ti_3_C_2_ with anticancer PTT (photothermal therapy) treatment abilities that lesser *in-vivo* toxicity than Ti_3_C_2_. The photothermal transformation capability of Au/Fe_3_O_4_/MXene and Au/MXene at the cellular level was assessed utilizing the cell line of breast cancer (MCF7). Behind incubation (with various concentrations of nanocomposites), evaluated the comparative viability of the cell without and with laser exposure. No apparent cytotoxicity was seen for “laser-free,” showing the high biocompatibility of nanocomposites (Figure figure6A). Also, Nanocomposites were subjected to a NIR laser (808 nm, 1.0 W/cm^2^) to assess the photothermal transformation performance for 5 min. According to Figure 6B, cell livability gradually reduced with the gaining concentration of both nanocomposites. Therefore, new nanocomposites can be more suitable and safer than Ti_3_C_2_ for biomedical applications, especially in the PTT method.

**FIGURE 7:**
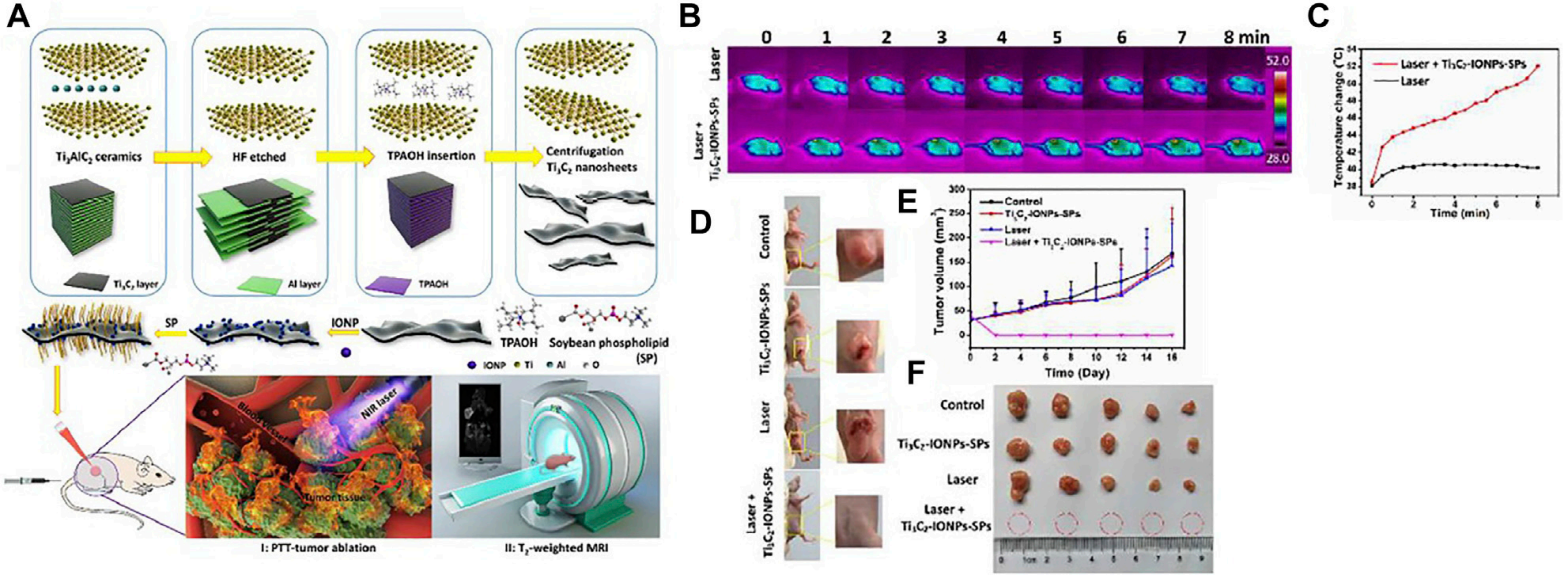
**(A)** Schematic of Ti_3_C_2_-IONPs-SPs synthesis and their multiple functions for tumor theranostics, **(B)** IR thermographic photographs of cancer mice afore and behind infusion of Ti_3_C_2_-IONPs-SPs nanocomposite followed by NIR radiance, **(C)** the temperature rise of tumors within the NIR radiance flow, **(D)** Digital images of mice with 4T1 tumor on the 16th day behind PTT therapies, **(E)** Tumor volume change plots of different groups of mice carriers of the tumor behind various therapies. **(F)** Collected digital images of tumors for every group at the PTT therapy end (Liu et al., 2018b).

In another study, Liu et al. (2018b) used magnetic MXenes for effective cancer therapy. The MXene is used by increased photothermal transformation ability for effective PTT versus cancer and IONPs action as a contrast factor for T_2_-weight MRI. Figure 7A displays synthesizing Ti_3_C_2_-IONPs nanocomposite and their particular theranostic function for cancer therapy. For the increased biocompatibility and stability of MXene-IONPs under physiological conditions, soybean phospholipids (Ti_3_C_2_-IONPs-SPs) were used. After Ti_3_C_2_-IONPs-SPs intravenous Injection, the tumor was irradiated straightly under the NIR laser (1.5 Wcm^−2^, 808 nm for 8 min). The tumor temperature variation was scanned via an IR thermal imaging camera monitored. Figures 7B,C displayed that the laser + Ti_3_C_2_-IONPs-SPs group temperature rose rapidly after NIR Laser radiance. But the Laser group temperature rose by only 2°C. The tumor disappeared considerably, and a black scar remained on the main sites of the tumor for the first days. However, other tumor groups constantly grew over a 16-days study period (Figures 7D–F). As a result, this nanocomposite Ti_3_C_2_-IONPs show high T_2_ relaxation of 394.2 mM^−1^s^−1^ and MRI with efficient tumor contrast, which provides the potential to conduct PTT. Ti_3_C_2_-IONPs have significant photothermal conversion efficiencies (48.6%) to decrease tumor tissue and kill cancer cells *in vitro* and *in vivo* (BALB/c nude and Kunming mice).

**FIGURE 8:**
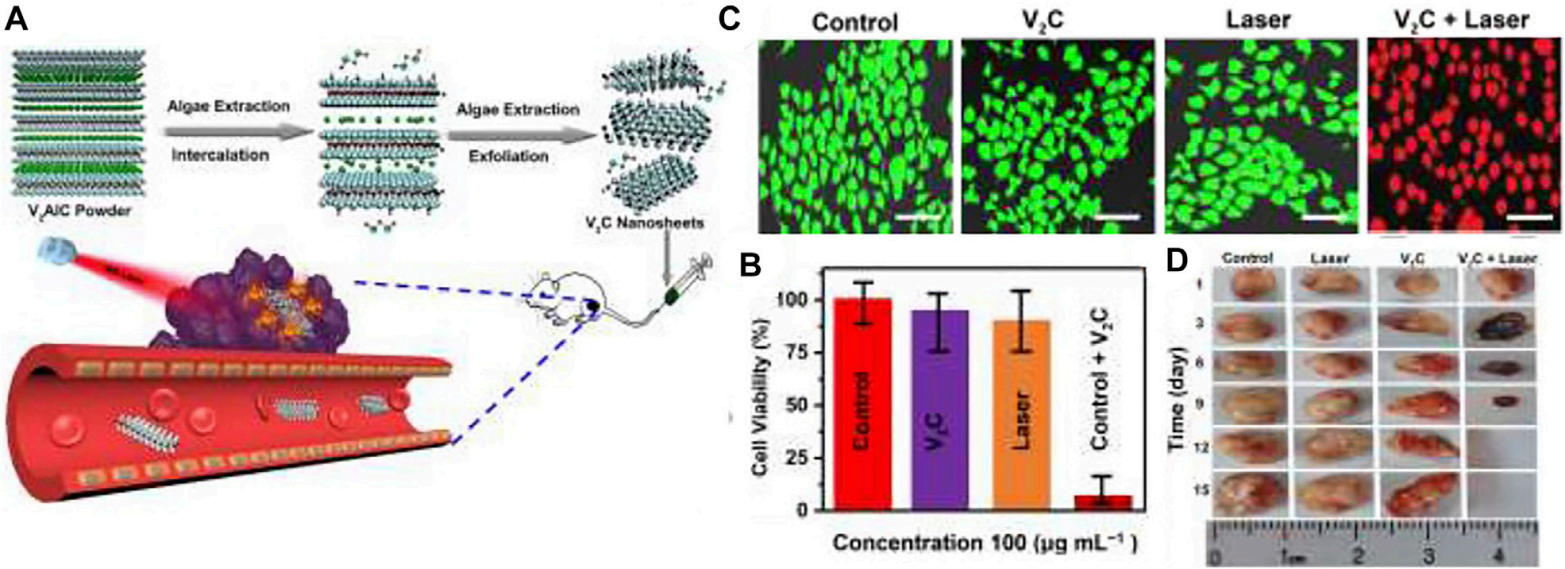
**(A)** Schematic representation of V_2_C-NSs synthesis and usages for PTT, **(B)** MTT method, and **(C)** result of Calcein AM/PI dual coloring of MCF-7 cell livability remedied with different treatment groups, **(D)** Tumor size photograph (Zada et al., 2020).

V_2_C (vanadium carbide) has well potential in the PTT method. Nevertheless, the usage of V_2_C in PTT is restricted due to difficult synthesis conditions and low PTCE. Zada and colleagues (Zada et al., 2020) developed a green synthesis way utilizing the extraction of algae to make V_2_C NSs for positively effectual *in-vivo* and *in-vitro* tumors photothermal ablation (Figure 8A). They investigated the effect of photothermal and bio-compatibility of V_2_C-NSs and anticancer function *in-vitro* on MCF-7 cells. Figure 8B shows that the V_2_C-NSs and Laser of NIR (0.48 W/cm, 808 nm, 10 min) alone showed partial toxicity. In contrast, V_2_C-NSs + Laser displayed considerable anticancer agents and murdered approximately all cells. Calcein AM/PI dual coloring analysis was compliance cum MTT outcomes (Figure 8C). These outcomes showed well *in vitro* anticancer efficacy of V_2_C-NSs owing to enhanced photothermal effectiveness. To check the anticancer efficacy of *in-vivo* studies, nude mice carrying MCF-7 tumors were separated into the control group (only PBS treatment), the Laser group, the V_2_C-NSs inject group, and the laser + V_2_C-NSs group. Laser + V_2_C-NSs combined therapy was considerably more efficacious in inhibiting tumor growth than the other groups. Finally, the tumor disappeared after 12 days (Figure 8D). Results demonstrated the excellent anticancer efficiency of V_2_C-NSs *in vivo*.

**FIGURE 9:**
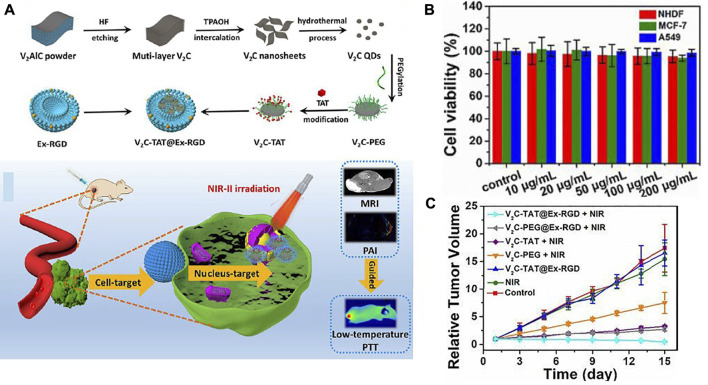
**(A)** The V_2_C-TAT@Ex-RGD preparation process and schematic of nuclear organ and cancer cell membrane with the dual objective of V_2_C-TAT@Ex-RGD nano-agent for PTT. **(B)** Check Cell viability of different cells (MCF-7, A549, and NHDF cells) were incubated at various concentrations V_2_C-TAT@Ex-RGD, **(C)** relative tumor growth plots in multiple groups (Cao et al., 2019).

The finite influence deepness of PTAs active in the NIR-I bio-window and thermal resistance induced by HSP considerably restrict the remedial effect of PTT. To solve this issue, Cao et al. (2019) introduced a PTT strategy for targeting (at down-temperature) nuclei in the NIR-II area that combines quantum dots of vanadium carbide (V_2_C-QDs) of PTA and Ex vector to kill effective tumors. Figure 9A shows the synthesis of V_2_C-QDs modified with Ex and TAT peptides (V_2_C-TAT@EX-RGD) with good thermal efficacy in the NIR-II region for PTT and good ability for MRI, fluorescent and photoacoustic imaging. The V_2_C-TAT@Ex-RGD *in vitro* cytotoxicity in different cells (MCF-7, NHDF, and A549) was evaluated using the MTT method. Figure 9B shows that V_2_C-TAT@Ex-RGD displayed partial toxicity to all cell lines, and cell viability was upper 90%. High bio-compatibility, excellent transmission performance, etc., make V_2_C-TAT@Ex-RGD an okay factor for PTT cancer. Seven groups were examined and displayed the plots of tumor growth (MCF-7 tumor) in Figure 9C. The group of control, V_2_C-TAT@Ex-RGD intinction, and Laser (1,064 nm, 0.96W/cm^2,^ and at 10 min) group have small suppressive efficacy upon tumor growth. V_2_C-PEG + Laser group, V_2_C-PEG@RGD + Laser, and V_2_C-TAT + Laser have little tumor growth. Notably, the V_2_C-TAT@Ex-RGD + Laser group showed substantial and effective suppression of tumor growth, and no recurrence occurred.

In Table 3, biocompatibility, photothermal conversion efficiency, and MXene nanosheet cell ablation effects on different types of breast cancer cells were investigated.

**TABLE 3:** Investigated biocompatibility, photothermal conversion efficiency, and the effect of MXene nanoplate cell ablation on different types of breast cancer cells.

Composition	Wavelength	Photothermal conversion efficiency	Cell line	Result/biocompatibility	Strategy	Refs
Au/MXene	NIR-I (Laser 808 nm, 1W/cm^2^)	—	MCF-7	*In-vivo* cytotoxicity measure utilizing zebrafish fetal displayed that Au/Fe_3_O_4_/MXene and AU/MXene had lower fetal murrain (LC» 1,000 µg/ml) than only MXene (LC = 257.46 µg/ml). Also, no apparent toxicity was observed for “without-Laser” indicating great bio-compatibility of nanocomposites	PTT	Hussein et al. (2019)
Au/Fe_3_O_4_/MXene						
Ti_3_C_2_-IONPs-SPs	NIR-I (Laser 808 nm, 1.5W/cm^2^)	48.6%	4T1	Ti_3_C_2_-IONPs-SPs have significant photothermal conversion efficiencies (48.6%) to decrease tumor tissues and kill cancer cells *in-vitro* and *in vivo* conditions	PTT	Liu et al. (2018b)
				For Nanocomposite (Laser-free), no displayed cytotoxicity was observed		
V_2_C-TAT@Ex-RGD	NIR-II (Laser 1,064 nm, 0.96W/cm^2^)	45.05%	MCF-7	Cell viability (>90%) for The V_2_C-TAT@Ex-RGD in different cells (MCF-7, NHDF and A549, *in vitro*)	PTT	Cao et al. (2019)
				The V_2_C-TAT@Ex-RGD + Laser group showed substantial and effective suppression of tumor growth, and no recurrence occurred (*in-vivo* method)		
V_2_C-NSs	NIR-I (Laser 808 nm, 0.48 W/cm^2^)	48%	MCF-7	Low toxicity in *in-vitro* method, V_2_C-NSs + Laser murdered approximately all cells (*in-vivo*)	PTT	Zada et al. (2020)
Nb_2_C-MSNs-SNO	NIR-II (Laser 1,064 nm, 1.5 W/cm^2^)	39.09%	HUVEC, 4T1	There is slight cytotoxicity to HUVEC and 4T1 cells, No chronic or acute response *in-vivo*. Optimal expulsion conduct, Nb_2_C-MSNs-SNO + Laser reduce tumor growth (*in-vivo*)	PTT	Yin et al. (2020)
Ti_3_C_2_-SPs	NIR-I (Laser 808 nm, 1W/cm^2^)	74.6%	4T1	#0D0D0D; Ti_3_C_2_ is a drug delivery (DOX) nano-platform for effective chemotherapy with great photothermal transformation ability of Ti_3_C_2_ for tumor deracination by photothermal ablation (both *in-vivo* and *in-vitro*), with No chronic or acute response *in-vivo*. Optimal expulsion conduct	PTT/chemotherapy	Han et al. (2018)
Ti_2_C-PEG	NIR-I (Laser 808 nm)	87.1%	MCF-7 non-malign MCF-10A	Fine bio-compatibility *in-vitro*, favorably effective cancer cell erosion, and well selectivity than malign cells	PTT/Photodynamic	Szuplewska et al. (2019a)
Nb_2_C-PVP	NIR-I (Laser 750–1,000 nm, 1W/cm^2^) and NIR-II (Laser 1,000–1,350 nm, 1W/cm^2^)	NIR-I = 36.4%	4T1	Nb_2_-PVP has little cytotoxicity (*in-vitro*) and great bio-compatibility	PTT	Lin et al. (2017)
		NIR-II = 45.65%		PPT ablation and tumor deracination (performance effective in both NIR-II and NIR-I, *in-vivo*)		
HAP/CS/HA/MXene	NIR-I (Laser 808 nm, 2 W/cm^2^)	HAP/CS/HA/MXene = 13.76%	MCF-7	Nanoplatforms have good bio-compatibility (*in-vitro*) and good photothermal transformation yields (*in-vivo*) with excellent potential for remote drug delivery (DOX)	PTT/drug delivery	Wu et al. (2021)
HAP/CS/HA/MXene/AuNRs		HAP/CS/HA/MXene/AuNRs = 20.42%				
Ti_3_C_2_-CoNWs	NIR-I (Laser 808 nm, 2 W/cm^2^)	34.42%	4T1	Ti_3_C_2_-CoNWs nanocarriers show great photothermal transformation efficiency under Laser radiance and excellent medicine loading capacity (DOX, 225.05%)	Chemo-PTT/drug delivery	Liu et al. (2020b)
H-Ti_3_C_2_-PEG	NIR-II (Laser 1,064 nm, 1 W/cm^2^)	Ti_3_C_2_ = 50.8% H-Ti_3_C_2_-PEG = 49.6%	4T1	Nanoplatforms have good biocompability and stability (*in-vitro* and *in-vivo*) and could improve the SDT performance	PTT and SDT	Li et al. (2022)
				It is important to note that H-Ti_3_C_2_-PEG is eliminated from the body. Furthermore, they arenot harmful long-term		

Due to the particular specifications of MXenes, these attractive features are crucial for their usages, such as biomedical (photothermal), antimicrobial use, and biosensors. Investigators have constructed prominent endeavors to develop synthesis and surface modification methods for diagnosis and treatment with photothermal for breast cancer. Detection and photothermal applications of MXenes are listed in Tables 2, 3 with surface profiles, limited detection, optical attributes, and conversion efficiency.

## 5 Coming prospects

The two-dimensional Mxene nanostructures are described in this study. But the rapid development of synthesized types of MXenes and their promising options for biomedical usage should be considered. Nanoplatforms based MXene on the response of small functional biomolecular, temperature, pH, and response should also be studied for probes and sagacious drug delivery so that diagnosis and effective therapy with fewer side effects can be achieved. Demonstrates a broad range of MXenes applications in the theragnostic of cancer, drug delivery, biosensors, and antimicrobial action that MXenes in early biomedical research may be believed to reduce or improve breast cancer.

## 6 Conclusion

This study provides an overview of the nanostructure of two-dimensional Mxene. It investigates different synthesis methods for producing biocompatible Mxenes and their application to the detection and therapy of breast cancer. In synthesis methods, a vast number of MXenes families are expected, while more recently, experimental species have been little demonstrated. MXenes’ surface modification is not only biocompatible but also has multifunctional properties, such as aiming ligands for preferential agglomeration at tumor sites for photothermal treatment that by the noncovalent reactions on the MXene surface with PEG, CS, SP, and PVP materials. The synthesized MXenes could modify to increase biodegradability/biocompatibility and decrease the cytotoxicity for particular biomedical usages. MXenes have a fantastic special for a great surface-to-volume proportion, antimicrobial attributes, drug delivery, engineering of tissue, and extensive near-infrared sorption. These features construct Mxenes as the most applicable materials for biological usage.
